# Development of a resilience-enhancing intervention during and after pregnancy: a systematic process informed by the behaviour change wheel framework

**DOI:** 10.1186/s40359-023-01301-4

**Published:** 2023-09-05

**Authors:** Sarah Van Haeken, Marijke A.K.A. Braeken, Antje Horsch, Mirjam Oosterman, Annick Bogaerts

**Affiliations:** 1 Research & Expertise, Resilient People, UC Leuven-Limburg, Wetenschapspark 21, 3590 Diepenbeek, Belgium; 2 Faculty of Medicine, department of Development & Regeneration, REALIFE Research Group, Women & Child KU Leuven, Leuven, Belgium; 3 Faculty of Rehabilitation Sciences, REVAL-Rehabilitation Research Center, Hasselt University, Diepenbeek, Belgium; 4 Institute of Higher Education and Research in Healthcare, University of Lausanne, Lausanne, Switzerland; 5 Department Woman-Mother-Child, Faculty of Biology and Medicine, Lausanne University Hospital, Lausanne, Switzerland; 6 Department of Clinical Child and Family Studies, Faculty of Behavioural and Movement Sciences, Vrije Universiteit Amsterdam, Amsterdam, the Netherlands; 7 Department of Nursing and Midwifery, CRIC Centre for Research & Innovation in Care, University of Antwerp, Antwerp, Belgium; 8 Faculty of Health, University of Plymouth, Devon, UK

**Keywords:** Psychological resilience, Perinatal Care, Primary Prevention, Public Health, Internet-based intervention

## Abstract

**Background:**

Pregnancy and the transition to parenthood are accompanied by multiple changes and stress exposure. Resilience has the potential to counteract the negative impact of stress and can be a protective factor against mental health problems. To date, the use of a theoretical framework in the development or application of resilience interventions during pregnancy up to one year postpartum is missing. The aim of this study is to develop an intervention to enhance resilience for pregnant women up to one year postpartum.

**Methods:**

A systematic and theory-based approach informed by the Behaviour Change Wheel framework and the theoretical model of perinatal resilience was applied. The development took place in three phases and during the process, the target group, researchers and clinicians were involved.

**Results:**

A combination of resilience-enhancing exercises, group sessions and an online support platform, including follow-up at six and twelve months after delivery, was designed to enhance resilience during pregnancy and up to one year postpartum. This intervention incorporates 5 intervention functions delivered by 18 behaviour change techniques.

**Conclusions:**

This study responds to the need for theory-based intervention programs aiming to enhance resilience to improve the psychological health of pregnant women. We developed a multicomponent resilience-enhancing intervention for pregnant women up to one year postpartum.

**Supplementary Information:**

The online version contains supplementary material available at 10.1186/s40359-023-01301-4.

## Introduction

Pregnancy and the transition to parenthood are potentially challenging for the pregnant woman and her partner involving changes on multiple levels that can affect their (mental) health following stress exposure [1]. Feelings of distress may be associated with biological (e.g., hormone fluctuations), physical (e.g., weight gain), social (e.g., change in social activities), and psychological changes (e.g., increased sense of responsibility) [2]. Stress has a substantial impact on the health and mental well-being of the individual [3]. Stress during pregnancy can have adverse effects on the woman herself, and can negatively affect pregnancy outcomes, infant health, postpartum mother-child interaction, and child development [3–5].

Characteristics of resilience and resilience-promoting mechanisms may counteract the negative impact of stress and have been linked to lower levels of anxiety and depressive feelings in the general population [6, 7]. Within the perinatal context, resilience is described as a multi-factorial construct influenced by individual, socio-cultural, and environmental factors [8]. A concept analysis and Delphi Survey of Van Haeken et al. (2020) [9] defined perinatal resilience for the first 1,000 days as:

Perinatal resilience is a circular process toward a greater wellbeing in the form of personal growth, family balance, adaptation, or acceptance, when faced with stressors, challenges, or adversity during the perinatal period. The presence of resiliency attributes such as social support, sense of mastery, self-efficacy, and self-esteem enhance the capacity to be resilient and prevent mental health problems (p. 9).

The perinatal period provides opportunities to promote mental health and resilience of future parents and, consequently, prevent the intergenerational transmission of stress and psychopathology in the next generation [10]. There is a clinical need for intervention programs aiming to enhance resilience by skill training and the application of evidence based tools [5, 11]. Previous meta-analyses of randomised and non-randomised controlled trials report positive intervention effects for increasing resilience in both clinical and non-clinical populations, such as college students and intensive care nurses [12, 13]. Although these interventions share the common aim to enhance resilience or resilience resources, they differ in terms of setting, outcome, content, format and length, which complicates evaluation [13–16]. Another limitation is the under-use of theoretical frameworks in intervention design [13, 14, 17]. The Medical Research Council (MRC) emphasizes the importance of using a theoretical framework in intervention development, resulting in interventions that are more likely to be successful [18]. The use of theory also promotes evaluation, making it possible to elucidate why and how different components of an intervention contribute to the overall effectiveness [19].

The Behaviour Change Wheel (BCW) framework [17] indicates a comprehensive, systematic and evidence-based stepped approach for intervention development. This framework is based on a synthesis of 19 theoretical frameworks of behaviour change pathways. Different studies on health promoting behaviour have already used the BCW to guide intervention design in a variety of health care settings including sexual counselling [20]. Within the perinatal context, the framework has been used with a focus on smoking cessation or breastfeeding behaviour [21]. Mostly, the focus of perinatal mental health interventions has been on risk assessment and the reduction of symptoms. However, mental health also includes a state of wellbeing and the capacity to cope with normal stress of life [22]. At present, however, a systematic, theory-based, development of an intervention to enhance resilience among pregnant women to prevent perinatal mental health problems is missing.

This paper describes a systematic and theory-based three-phase approach of intervention development. Intervention design was informed by the recommendations of the BCW framework for developing complex behaviour change interventions and the theoretical model of perinatal resilience.

The aim of the present study was to develop an intervention to enhance resilience during pregnancy with follow-up till one year postpartum.

## Method

This study consisted of three phases: (1) identifying relevant COM-B (i.e. Capabilities, Opportunities, Motivations, Behaviour) components, (2) identifying intervention functions, (3) identifying and prioritising behaviour change techniques (BCTs) and modes of delivery. The development process is reported following the Guidance for Reporting Intervention Development Studies in Health Research (GUIDED) checklist, consisting of 14-item quality criteria (Additional file 1) [23]. The study received ethical approval from the Ethics Committee of University Hospital/Catholic University of Leuven, Belgium (registration number B322201940153) and the Commission Medical Ethics Hospital Oost-Limburg, Genk, Belgium (registration number B371202042785).

### Phase 1: identifying relevant COM-B components

The first phase aimed to identify resources mothers use and/or need to cope with stress and promote resilience during pregnancy and after childbirth. The COM-B model as central part of the BCW framework, helps to capture volitional behaviour by the assumption that for someone to engage in a particular behaviour (B), they must be physically and psychologically capable (C), have the social and physical opportunity (O), and want, or need to perform the behaviour out of an automatic and reflective motivation (M) [24]. The COM-B components i.e., capability, opportunity, and motivation, were explored by semi-structured individual interviews.

#### Sample

Participants were recruited in a semi-residential infant mental health facility in a psychiatric centre in Belgium [25]. This facility focuses on families of infants with persistent regulatory and/or developmental problems. This choice was made to identify the needs for resilience support as well as the behaviours that can be assessed to enhance resilience in a stressed population with their resilience under pressure. Purposive sampling was used to ensure participants met the inclusion criteria. These were: (1) being admitted to outpatient services of the infant mental health facility between 2016 and 2019 with an infant aged 1 to 24 months; (2) minimum age of 18 years; and (3) sufficient fluency in spoken Dutch. The exclusion criteria were as follows: (1) pre-existing bipolar and/or psychotic disorder; and (2) diagnosed depressive or anxiety disorder at the time of recruitment for the interview after discharge from the infant mental health facility. Based on these criteria, 21 out of 42 mothers admitted to the infant mental health services were eligible. Of these, 13 mothers gave consent to take part in the study [25].

#### Instruments

A comprehensive interview guide was used containing specific questions framed by the theoretical model of perinatal resilience. This model defines the antecedents, consequences, and main attributes of perinatal resilience as being social support, self-esteem, self-efficacy, sense of mastery, and personality [9]. The main questions in the interview revolved around three themes: (1) factors that promote or suppress perinatal resilience, (2) needs related to perinatal resilience, and (3) desired fulfilment of these needs. All interviews were audio recorded and transcribed ad verbatim. An exploratory qualitative research design was adopted, using the Qualitative Analysis Guide of Leuven (QUAGOL) [26]. The QUAGOL, a method inspired by the grounded theory approach, was chosen to reconstruct the story of the participants at a theoretical level and to analyse the concepts found [27]. The conceptual coding scheme was developed following the three COM-B categories. In the first round of coding, each experience and statement was coded according to one of the three COM-B categories and converted into a list of concepts. A constant comparative analysis was used in which concepts were compared with each other, within one interview and between different interviews. All concepts used were listed, evaluated, and discussed by members of the research team (SVH, MB, AB). For further details on the recruitment, data collection, and analysis, we refer to the study of Nuyts et al. (2021) [25], since this phase was part of a broader research project.

### Phase 2: identifying intervention functions

In phase two, the relevant intervention functions were identified. The BCW framework proposes nine intervention functions: education, persuasion, incentivisation, coercion, training, enablement, modelling, environmental restructuring, and restrictions [17]. The effectiveness of the intervention function is related to the COM-B component for which it will be deployed. For example, an intervention function may be very effective for strengthening the capabilities component of the COM-B model but have less impact on strengthening opportunities. The identified COM-B components regarding perinatal resilience (phase 1) were mapped onto the published BCW linkage matrices (Additional file 2) that link each COM-B component to the most effective intervention function [24]. Additionally, the predefined intervention functions were assessed in a nonblinded expert panel consensus meeting (N = 15). This panel consisted of researchers (N = 8) and clinicians (N = 7). Some experts were already part of the project’s steering committee. Others were invited via network sampling because of their expertise on the concept of resilience, intervention development and/or implementation within a perinatal context. The expert panel met in October 2019 to discuss preliminary findings and the initial intervention development process generated by the research team. First and second author (SVH & MB) led the session and the last author (AB) moderated the group discussion.

### Phase 3: prioritising BCTs and identifying modes of delivery

In the third phase, specific behaviour change techniques (BCTs) and effective modes of delivery were identified [28]. A BCT is defined as “an observable, replicable, and irreducible component of an intervention designed to alter or redirect causal processes that regulate behaviour” [28]. The Behaviour Change Technique Taxonomy (BCTTv1) comprises 93 techniques [24, 29]. The BCW guidelines previously established relevant associations between intervention functions and BCTs and were used to assist with the selection of BCTs. In addition, we reviewed the recent literature on intervention development and organised a follow-up expert panel consensus meeting (N = 17) in September 2020 to review the suitability of BCTs, in the light of the findings of the previous phases. The panel consisted mainly of the same individuals as the first round, complemented by one researcher and one clinician working in the perinatal context.

## Results

Subsequently, the findings are bundled into the operationalisation of an intervention which aims to enhance resilience.

### Phase 1: identifying relevant COM-B components

**Fig. 1:**
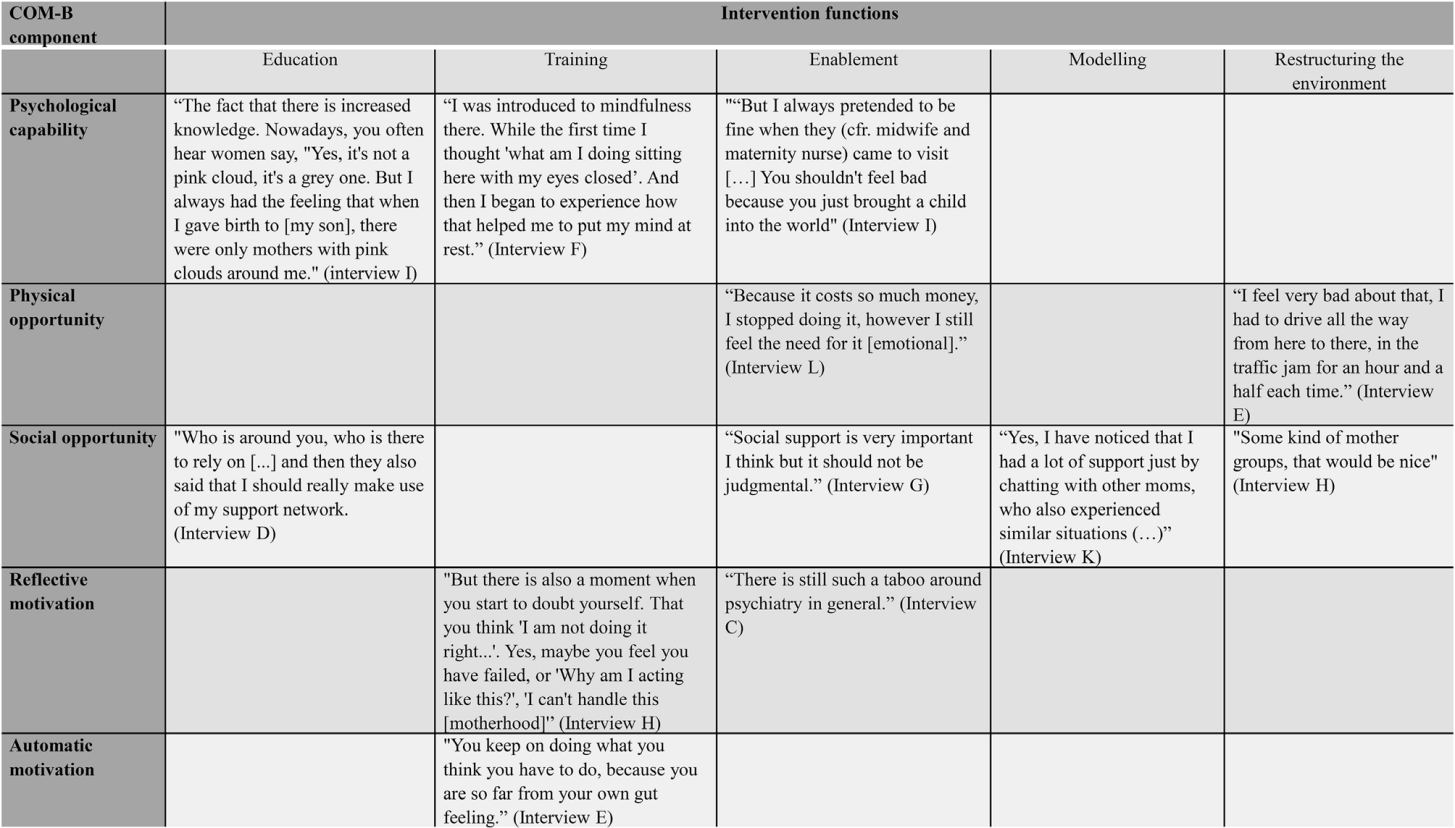
Matrix of COM-B components and intervention functions

The first phase aimed to identify resources mothers use and/or need to cope with stress and promote resilience during pregnancy and after childbirth. The COM-B components were explored by thirteen face-to-face individual semi-structured interviews. All mothers were Caucasian, born in Belgium and their mean (SD) age at the time of the interview was 33.6 (4.6) years. Ten mothers lived with a partner, three mothers were divorced or no longer living with the father of their child. The majority of mothers had at least a bachelor’s degree (N = 11) and were employed (N = 8). The infant’s mean age (SD) at the time of admission was 10.9 (5.2) months. The mean duration of admission was almost 6 (5.9, SD 2.0) months. Based on the qualitative analyses, following COM-B components were identified: capability (psychological), opportunity (physical and social), and motivation (reflective and automatic) (Fig. 1). Physical capability was not identified.

#### Capability

The first component of the COM-B model is capability, which states that people must have the physical or psychological strength to perform the behaviour. Based on the interviews with mothers, we could distinguish two factors in relation to psychological capability: knowledge and psychological skills.

A first factor is *knowledge* about perinatal mental well-being. Mothers thought that pregnancy and childbirth were perceived by society as moments of happiness with no space for negative feelings. Participants described this as being on a ‘pink cloud’ (also known as cloud nine, a state of perfect happiness), which was sustained in part by general opinions in society, social media, and television channels. Many mothers had a contrary experience to what they had expected and experienced negative feelings. Improved understanding of the impact of perinatal mental health problems on family functioning, resilience and stress(systems) may help to counter the perception of a ‘plink cloud’.

*“The fact that there is increased knowledge. Nowadays, you often hear women say, “Yes, it’s not a pink cloud, it’s a grey one. But I always had the feeling that when I gave birth to [my son], there were only mothers with pink clouds around me”. (interview I)*

A second factor is *psychological skills* containing coping resources and emotion regulation. The psychological toll of becoming a parent can be tough. Participants experienced negative feelings contradicting the anticipated feelings of happiness. To cope with their feelings, some mothers held up a facade and presented themselves in a way they thought others were expecting them to behave. Another coping strategy mentioned by some mothers was an escape into work or sleep medication.

*“But I always pretended to be fine when they (cfr. midwife and maternity nurse) came to visit […] You shouldn’t feel bad because you just brought a child into the world” (Interview I)*.

Mothers felt like they could no longer control the situation and they experienced a lack of connection with their own and their infant’s emotions. Mothers got stuck in a vicious cycle, inhibiting their own feelings and desires. This led to difficulties within the mother-infant interaction from not understanding the child’s signals to not feeling like a loving parent.

*“You let yourself get carried away so quickly by an emotion that you don’t longer see it anymore.” (Interview J)*.

Participants emphasised the importance of their own emotion regulation as a significant component of perinatal resilience. Being able to be aware of their emotions, to recognise them and to put them into perspective, enhances their sense of resilience. However, mothers expressed the need to develop the skills, which allow them to be aware, recognise and put their emotions into perspective, such as mindfulness and relaxation to enhance their emotion regulation capabilities.

*“I was introduced to mindfulness there. While the first time I thought ‘what am I doing sitting here with my eyes closed’. And then I began to experience how that helped me to put my mind at rest.” (Interview F)*.

Mothers mentioned self-care, looking after themselves, as an important coping strategy in times of stress. For example, trying to relax, practising hobbies (e.g., sports) as well as getting enough sleep appeared to be important. A lack of sleep was mentioned as an important factor that negatively influenced the well-being of mothers.

*“I think that that [sleep deprivation] took away a lot of my resilience, because I was so exhausted” (Interview M)*.

#### Opportunity

The second component is opportunity, which represents the physical and social opportunity for behaviour change to occur. Based on the interviews with mothers, we could distinguish two factors in relation to physical opportunity: accessibility and affordability of resources. Social opportunity involves interpersonal influences, social cues, and cultural norms that influence the way we think.

Mothers attach great weight to an *easy accessibility* of support programs. An extra support program needs to fit within the numerous consultations within the standard care pathway, their work commitments, and the busy schedule if they already have children. Time and place are important factors to consider in the development of a support program. If the burden is too high, this is an important reason to reject or stop attendance at a support program.

*“I feel very bad about that, I had to drive all the way from here to there, in the traffic jam for an hour and a half each time.” (Interview E)*.

Another pitfall is the *affordability of resources*. Psychological support is associated with high costs, making it for some mothers not affordable.

*“Because it costs so much money, I stopped doing it, however I still feel the need for it [emotional].” (Interview L)*.

A factor of social opportunity is the availability of a *supportive social network.* Mothers emphasize the importance of social support as part of resilience and as a protective factor that buffers the negative influences of stress. Important sources of support are the partner, family members, and the broader network (e.g., friends, colleagues). They can support (future) parents at various levels such as practical, informative and emotional support. Despite the importance of social support, mothers reported barriers in engaging their social support network regarding childcare or household duties. They viewed a potential request for help as indicator of not being able to handle parental responsibilities.

*“In retrospect, that was stupid of me too because I was just way too proud to ask for help.“ (Interview I)*.

Mothers therefore mentioned that they needed to be actively encouraged to reach out to their network for help.

*“Who is around you, who is there to rely on […] and then they also said that I should really make use of my support network. (Interview D)*

An important need regarding social support is the need to be heard without judgment. Mothers indicated that the desired support consisted mostly of the recognition and confirmation of their feelings.

*“We felt heard, and at ease and reassured about ‘it’s not abnormal, you’re not the only one” (Interview E)*.

*“Social support is very important I think but it should not be judgmental.” (Interview G)*.

The support of health care professionals was highly valued, where mothers appreciated continuity of care, consistent advice, and attention to the psychological aspects of becoming a parent such as the shift in the couple relationship. The absence of professional support caused feelings of despair. Also, the lack of attention to the mental health of pregnant women or new mothers frustrated participants.

*“If you don’t get help and you don’t get support, there comes a moment when you feel so desperate” (Interview G)*.

The support of peers was highlighted by mothers in the interviews. Peers go through similar experiences, which may lead to feelings of understanding and acknowledgement. Understanding that other new mothers have similar experiences can be empowering and can strengthen the confidence of mothers to seek out solutions in the knowledge that she is not alone. One way to access peer support is through participating in mother groups.

*“Yes, I have noticed that I had a lot of support just by chatting with other moms, who also experienced similar situations (…)” (Interview K)*.

#### Motivation

The third component is motivation, which is divided into reflective and automatic motivation [17]. Based on the interviews with mothers, we could distinguish two factors in relation to reflective motivation: self-efficacy and stigma. Automatic motivation involves emotional reactions, desires, and reflex responses.

*Self-efficacy* can be categorized under reflective motivation since it is the mother’s ability to evaluate and reflect on their skills and if they are sufficient to perform the necessary behaviour [30]. Mothers frequently experienced feelings of doubt and uncertainties regarding their early parenthood, putting their resilience under pressure.

*“But there is also a moment when you start to doubt yourself. That you think ‘I am not doing it right…’. Yes, maybe you feel you have failed, or ‘Why am I acting like this?‘, ‘I can’t handle this [motherhood]’’ (Interview H)*.

Mothers indicated that they had to learn to trust themselves and their own competence. Being confirmed in their role as a mother by their support network and being encouraged by others was considered important.

Another component of reflective motivation is the pre-existing *stigma* regarding perinatal mental health problems. Mothers recognised it in themselves and in the caregivers. As a result, they doubted whether psychological support was appropriate because they linked this kind of support to being ‘crazy’.

*“There is still such a taboo around psychiatry in general.” (Interview C)*.

In case of automatic motivation, mothers emphasized the importance of their *gut feeling*.

*“You keep on doing what you think you have to do, because you are so far from your own gut feeling.” (Interview E)*.

To conclude from the interviews, knowledge, psychological skills, social support, and self-efficacy have been identified as important resources for mothers in case of perinatal resilience. Barriers to take into account refer to the accessibility and affordability of resources and the pre-existing stigma regarding perinatal mental health problems. In the following phases, the COM-B components were linked to the intervention functions and behaviour change techniques.

### Phase 2: identifying intervention functions

In the second phase, the identified COM-B components were linked to the relevant intervention functions. Following Michie et al. (2014) [24], complex interventions could have multiple functions and the selection of the functions requires judgement of what is most appropriate for the context. Out of the nine proposed intervention functions by the BCW framework, five were considered suitable (Fig. 1) based on published BCW linkage matrices (Additional file 2), mothers’ report (phase 1), and an expert panel consensus meeting. Coercion, persuasion, and restriction were rejected as unsuitable, as these go against an approach focusing on resilience. Education, training, and enablement were considered as main functions of the intervention.

### Phase 3: prioritising BCTs and identify modes of intervention delivery

**Fig. 2:**
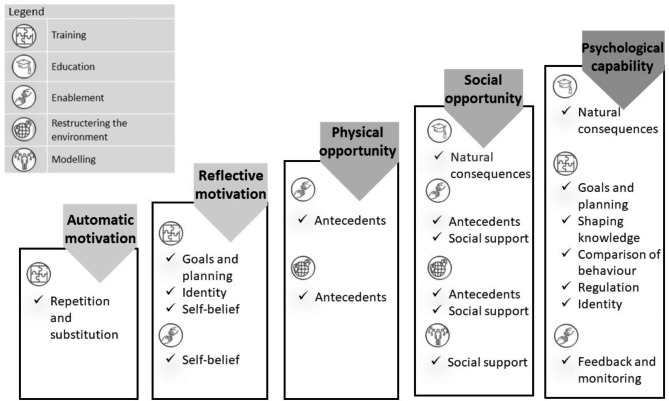
Overview of the COM-B components, intervention functions and BCT groupings

During the last phase, the content of the intervention was selected in terms of potential behaviour change techniques appropriate for each selected intervention function. A BCT is an active component of an intervention designed to change behaviour and is applicable to a range of different health behaviours. For each intervention function, the BCW guide lists the most and less frequently used BCTs according to the Behaviour Change Techniques Taxonomy version 1 (BCTTv1) [28]. This taxonomy consists of 93 items that can be divided into sixteen groups of techniques. This was used to facilitate the selection of relevant BCTs. In total, the research team selected 18 BCTs to be included in the intervention. These are associated with the following BCT grouping: goals and planning, feedback and monitoring, social support, shaping knowledge, natural consequences, comparison of behaviour, repetition and substitution, regulation, antecedents, identity, and self-belief. After a first selection by the research team (SVH, MB, AB), the selection was discussed within a follow-up expert panel consensus meeting. Following criteria; (1) affordability, (2) practicability, (3) acceptability, and (4) equity (Michie et al., 2014) were discussed, evaluated, and taken into account for the final selection of BCTs and their operationalisation. The chosen BCT groupings were linked to the selected COM-B components and intervention functions obtained in phase 2 (Fig. 2). Details about the individual BCTs and how they were operationalised in a resilience-enhancing intervention are presented in additional file 3.

#### Mode of intervention delivery

At last, the mode of intervention delivery was decided [17]. Modes of delivery can be broad, such as delivery at distance or face-to-face on individual, group or population level. Based on the interviews with the mothers (phase 1), physical and social opportunities seemed important to take into account. Physical opportunity relates to accessibility and affordability of the intervention. Pregnant women face barriers such as busy schedules, because of continued work and regular consultations with health care providers during pregnancy. Also, during the immediate postpartum, mothers find it often difficult to leave the house due to their recovery after childbirth, the lack of daily structure, and the demands of infant care. In response to this needs, an online format was selected as the primary mode of intervention delivery. Online interventions may be particularly appealing for mothers because of the flexibility and time efficiency, making it possible to follow and complete the program anytime and anywhere.

. Interactive online environments, such as Facebook, blogs, and smartphone applications (apps) are also popular among mothers [31]. Another important finding of phase 1 is the importance of social- and peer support (social opportunities). Therefore, it was proposed to deliver the intervention in a group format and to develop an online support platform. To enhance the effectiveness and adherence of the online intervention, human support by a psychologist, midwife and peers will be applied throughout the intervention.

#### Operationalisation

**Fig. 3:**
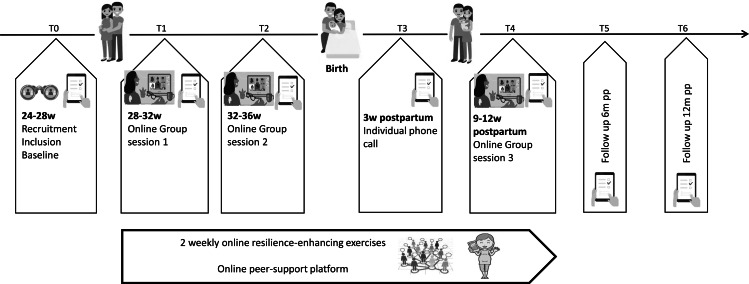
Operationalisation of the developed intervention

After a systematic intervention development process, a 28-week online perinatal program aimed to enhance resilience was constructed. The program consists of resilience-enhancing exercises, group sessions, and an online peer-support platform (Fig. 3). Through these components, the intervention aims to address the needs of mothers regarding perinatal resilience in terms of knowledge, psychological skill training, self-efficacy, and social support.

The program starts during pregnancy and continues up to twelve weeks postpartum, with two follow-ups at 6 months and one year postpartum. At enrolment, women will be grouped according to their gestational age. Group size was maximised to six participants plus two counsellors (clinical psychologist/midwife) trained in the content and the delivery of the program. Women will be invited for a first online group session at 28–32 weeks of pregnancy, after which they get access to resilience-enhancing exercises and an online peer-support platform in the format of confidential Facebook groups, where they can exchange experiences and support each other. This online intervention comprises different forms of human-support being: counselling by a psychologist and midwife during the online group sessions, an individual phone call to the participant around three weeks after childbirth and peer-support by the online platform. In addition, personalised online information with tailored messages depending their gestational age or postpartum duration were shared on the online platform.

## Discussion

This study describes the development of a resilience-enhancing intervention during pregnancy and up to one year postpartum. The theoretical framework of perinatal resilience [9] in combination with the BCW framework guidelines [24], provides a systematic approach to the development of a targeted intervention.

Based on previous research and the framework of perinatal resilience [9], we can state that resilience is a multifactorial concept [8]. Our newly developed intervention is thus complex, and consists of several interacting components. The identified COM-B components were linked to five intervention functions and eighteen BCTs. The chosen functions and BCTs are in line with current evidence. Beard et al. [32] studied 69 papers and 15 reviews, covering 251 health behaviour change interventions. Those interventions were specified in terms of their intervention functions and their BCTs. The most prevalent functions in health behaviour change interventions (N = 251) were training (74.9%), education (72.1%), and enablement (45.8%). A quarter of the interventions studied used persuasion, although those interventions were more likely to address addictive behaviour. Approximately 5% of the interventions incorporated the functions environmental restructuring and incentivization. Restriction (2.8%), modelling (1.2%), and coercion (0%) were rarely or never used [32]. The selected BCT groupings were also particularly prevalent in previous interventions: shaping knowledge, antecedents, regulation, and social support. About half of the interventions considered one or more of these BCT groupings [32]. A further five BCT groupings were commonly identified: comparison of outcomes, feedback and monitoring, goals and planning, natural consequences, and self-beliefs. They were evident in a fifth to a third of interventions. Instructions on how to perform a behaviour (part of shaping knowledge) was reported in nearly nine out of ten interventions [32]. Previous interventions designed to enhance resilience commonly involved psychoeducation, cognitive-behavioural components (e.g. cognitive reframing), skill training, and peer-support [33].

A systematic review by Scope et al. (2017) [34] suggests that how information is provided, is important because of the existing stigma regarding perinatal mental health problems and asking for this type of information. Realistic information about common experiences can promote more informed expectations about the changes that come with the transition to parenthood, which supports resilience [25]. There is a need for prenatal programmes that aim to promote resilience by stimulating existing protective factors and taking into account women’s perspectives on resilience and perinatal mental health. These programmes go beyond an individual trait approach and explore the many contextual, social and economic determinates of resilience [35]. Those programmes are also likely to have the greatest long-term impact [10]. Given the positive emotions automatically associated with childbirth, women sometimes feel limited to share their negative emotions. We need to enable women to express a diversity of emotions by creating a space where women can speak openly, feeling safe, and without facing prejudice. Practising mindfulness may be a useful skill and a powerful approach to reduce prenatal maternal stress. Mindfulness-based interventions have the potential in the perinatal period both to prevent the onset of and to alleviate existing mental health difficulties [36].

Online delivery was chosen as the primary mode of delivery. Previous research demonstrated that the use of online resources, and particularly mobile devices, shows significant promise for supporting health behaviour change [37, 38]. Mothers often seek participation in electronic support groups for information and advice to navigate and deal with challenges during and after pregnancy [39]. The use of a digital medium, however, does not imply an impersonal approach. The developed intervention combined a human support component at different time points to enhance the effectiveness and adherence. Research of Santarossa et al. (2018) [40] shows that implementing online human-supported interventions, can foster results similar to a face-to-face intervention. Another important element, certainly in the perinatal period, is peer support. Research shows that peer support groups can offer a safe and destigmatising environment, which helps new mothers to understand and deal with changes during pregnancy and the postpartum period [41]. Perinatal peer support interventions also have the potential to improve mental health outcomes and to decrease the risk of the development of postpartum depression [41]. Another evolution is the focus on the maintenance and promotion of positive health and well-being during pregnancy. Routine care focuses mostly on clinical detection and treatment of potential or actual pathology [42] on an individual level. Given the long waiting lists and the cost of mental health care [43, 44], a digital, easy accessible, and low-cost prevention program is urgently needed.

### Strengths and limitations

A strength of this study is the theoretical and systematic approach informed by the guidelines of the BCW framework and the model of perinatal resilience, resulting in a transparent report of the intervention development. However, despite the BCW guidelines, the selection of intervention functions and BCTs involves personal judgement and a certain amount of subjectivity. Therefore, triangulation of evidence was used to guide decisions by conducting in depth interviews with the target group, organising two expert panel meetings and integration of current evidence regarding health behaviour change interventions. In addition, the research team had a multidisciplinary background (health, developmental and clinical psychology, midwifery) which enabled a holistic decision making process. The interaction between the multidisciplinary research team and the expert panel resulted in a feedback loop at multiple stages of the intervention development, maximising the potential of success and effectiveness of the intervention when implemented in clinical practice. However, some limitations should be addressed. First, the BCW framework informed the development process but wasn’t applied in its entirety. A behavioural diagnosis was not applied and policy categories were not identified. Second, the COM-B model which can be applied to any behaviour and is simple to understand, has two major challenges: the broadness of the components and their inter-relatedness. The presence of one component can lead onto the presence of others. For example, a broad social network and interpersonal skills can enable mothers to ask people for help, giving them ‘time’ for self-care. Another difficulty is the influence of motivation to behaviour, which is complex. The COM-B model separates reflective and automatic motivation. However, automatic motivation is difficult to interview and less likely to be discussed and talked about spontaneously. Third, the interviews were only conducted with mothers whose resilience was under great pressure during pregnancy and/or the postpartum period. Finally, despite the possibility of reaching a large, geographically widespread, group of women using an online intervention, the difficulty of reaching a diverse group regarding socio-economic background needs to be taken into account. Moreover, women must also have access to online devices to participate in the programme.

### Future research

Following the Medical Research Council guidance [44], the resilience-enhancing intervention described in this study will be piloted in a feasibility study. The feasibility study aims to assess the potential effectiveness, accessibility, and acceptability of the developed intervention within a community sample of pregnant women. In a next step, a randomised controlled trial (RCT) aimed to evaluate the effectiveness of the intervention developed to enhance resilience during and after pregnancy on the prevention of perinatal mental health problems will be conducted.

## Conclusion

In this study, we executed a systematic and comprehensive development of a multicomponent intervention to enhance resilience during pregnancy and up to one year postpartum informed by the BCW framework and the theoretical model of perinatal resilience. Based on the experiences of mothers, different COM-B components supported a targeted intervention. In total, we identified intervention functions to enhance perinatal resilience, with ‘education’, ‘training’, and ‘enablement’ being the most relevant ones. For each intervention function, one or more behaviour change techniques (BCTs) were selected, leading to a total of eighteen BCTs. The operationalization of the BCTs led to a 28-week online intervention program consisting of group sessions, resilience-enhancing exercises, and an online peer support platform, aiming to enhance resilience and to prevent perinatal mental health problems. In the next phase, this intervention will be tested in a feasibility study.

### Electronic supplementary material

Below is the link to the electronic supplementary material.

Supplementary Material 1

Supplementary Material 2

Supplementary Material 3

## Data Availability

The datasets used and/or analysed during the current study are available from the corresponding author on reasonable request.

## Abbreviations

BCT(s): Behaviour Change Technique(s)
BCTTv1: Behaviour Change Technique Taxonomy version 1
BCW: Behaviour Change Wheel
COM-B: Capability, Opportunity and Motivation model of Behaviour
GUIDED: GUIDance for rEporting of intervention Development
RCT: Randomised Controlled Trial
QUAGOL: Qualitative Analysis Guide of Leuven
MRC: Medical Research Council
