# Supplementary material for: Development of a resilience-enhancing intervention during and after pregnancy: a systematic process informed by the behaviour change wheel framework
Source: BMC Psychol. 2023 Sep 5;11:267. doi: 10.1186/s40359-023-01301-4 (PMC10481562; doi:10.1186/s40359-023-01301-4)
Supplement: Supplementary file 2 — Supplementary Material 2 [file 40359_2023_1301_MOESM2_ESM.pdf]

Additional file 3: 18 selected behaviour change techniques (BCTs) for a perinatal resilience enhancing intervention

**BCT Label**<br>**Definition** (Michie et al., 2013)	**Intervention strategy**
Problem solving (1.2)<br>Analyse, or prompt the person to analyse, factors influencing the behaviour and generate or select strategies that include overcoming barriers and/or increasing facilitators.	Participants are trained to identify specific triggers that cause stress and to develop strategies to reduce/manage stress and negative emotions.
Action planning (1.4)<br>Prompt detailed planning of performance of the behaviour (must include at least one of context, frequency, duration and intensity). Context may be environmental (physical or social) or internal (physical, emotional or cognitive; includes 'Implementation Intentions').	Participants receive self-management exercises, which they can perform at a particular time on certain days of the week.
Feedback on behaviour (2.2)<br>Monitor and provide informative or evaluative feedback on performance of the behaviour (e.g. form, frequency, duration, intensity).	Participants have the opportunity to request feedback regarding their resilience scores in order to efficiently focus on self-management exercises. To motivate participants in their progress, they received feedback on their behaviour during the group sessions, and discuss barriers regarding perinatal resilience and how to overcome them.
Social support (3)<br>Advise on, arrange or provide social support (practical, emotional) from friends, relatives, colleagues, peers,…. Non-contingent praise or reward for performance of the behaviour.	Participants will be encouraged to map their social network and actively seek help. Support can include practical (e.g., child care), emotional (e.g., listening), affirmational (e.g., support aimed at promoting self-esteem and self-confidence), and/or informational support. In addition, participants are encouraged to support each other.
Instruction on how to perform the behaviour (4.1)<br>Advise or agree on how to perform the behaviour (includes 'skills training').	Participants receive instructions on how to effectively perform the self-management exercises. During the group sessions, their skills are trained.
Information about health consequences (5.1)<br>Provide information (e.g. written, verbal, visual) about health consequences of performing the behaviour.	Participants receive information about the explicit benefits of perinatal mental well-being and resilience during and after pregnancy for the mother, her infant, and the partner, e.g., physical and mental health, cognitive development, and bonding.
Monitoring of emotional consequences (5.4)<br>Prompt assessment of feelings after attempts of performing the behaviour.	Participants will record their feelings after completing the self-management exercises.
Information about emotional consequences (5.6)<br>Provide information (e.g. written, verbal, visual) about emotional consequences of performing the behaviour.	Participants receive information about the influence of the self-management exercises on perinatal mental well-being and resilience.

Demonstration of behaviour (6.1)<br>Provide an observable sample of the performance of the behaviour, directly in person or indirectly e.g. via film, pictures, for the person to aspire to or imitate.	Participants receive a demonstration on how to perform the self-management exercises and relaxation exercises.
Behavioural practice/rehearsal (8.1)<br>Prompt practice or rehearsal of the performance of the behaviour one or more times in a context or at a time when the performance may not be necessary, in order to increase habit and skill.	Participants receive the message to practice the relaxation exercises daily and are prompted to practice
Generalisation of target behaviour (8.6)<br>Advise to perform the wanted behaviour, which is already performed in a particular situation, in another situation.	Participants receive the advice to repeat relaxation exercises learned during the group session when at home.
Reduce negative emotions (11.2)<br>Advise on ways of reducing negative emotions to facilitate performance of the behaviour.	Participants are advised to use stress management skills and self-management exercises to reduce stress and negative emotions (e.g., frustration, depressive feelings, anxiety)
Restructering physical environment (12.1)<br>Change, or advise to change the physical environment in order to facilitate performance of the wanted behaviour or create barriers to the unwanted behaviour.	The program creates the opportunity to join a peer-support platform and group sessions.
Restructering social environment (12.2)<br>Change, or advise to change the social environment in order to facilitate performance of the wanted behaviour or create barriers to the unwanted behaviour.	Participants receive the advice to map their social network and are prompted to explicitly ask help when needed.
Framing/reframing (13.2)<br>Suggest the deliberate adaptation of a perspective or new perspective on a behaviour in order to change cognitions or emotions about performing the behaviour.	Negative thoughts are often exaggerated and do not enhance perinatal resilience. Participants are instructed to redirect non-helpful thoughts into helpful ones.
Valued self-identity (13.4)<br>Advise the person to write or complete rating scales about a cherished value or personal strength as a means of affirming the person's identity as part of a behaviour change strategy.	Participants receive the advice to write about their personal strengths and accomplishments. In addition, participants will ask persons from their own social network to write down a positive self-portrait about the person.
Verbal persuasion about capability (15.1)<br>Tell the person that they can successfully perform the wanted behaviour, arguing against self-doubts and asserting that they can and will succeed.	Participants will be encouraged that they can successfully increase their resilience.
Focus on past success (15.3)<br>Advise to think about or list previous successes in performing the behaviour (or parts of it).	Participants receive the advice to describe or list their achievements or events they consider as successful.
