# Supplementary material for: Development of a resilience-enhancing intervention during and after pregnancy: a systematic process informed by the behaviour change wheel framework
Source: BMC Psychol. 2023 Sep 5;11:267. doi: 10.1186/s40359-023-01301-4 (PMC10481562; doi:10.1186/s40359-023-01301-4)
Supplement: Supplementary file 3 — Supplementary Material 3 [file 40359_2023_1301_MOESM3_ESM.pdf]

Additional file 2: Matrix of links between COM-B components and intervention functions developed by Michie et al. (2014)

**COM-B Components**	**Intervention types**								
	Education	Persuasion	Incentivisation	Coercion	Training	Restriction	Environmental restructuring	Modelling	Enablement
Physical capability					X				X
Psychological capability	X				X				X
Physical opportunity					X	X	X		X
Social opportunity						X	X	X	X
Automatic motivation		X	X	X	X		X	X	X
Reflective motivation	X	X	X	X					

X indicates the intervention types most applicable for bringing about change in each COM-B component

Source: Michie et al. (2014)
